# Debilitating Chronic Diarrhea Caused by Generalized Gastrointestinal Cytomegalovirus Infection in an Immunocompetent Adult

**DOI:** 10.1155/2014/260120

**Published:** 2014-06-12

**Authors:** Emmanouil Telakis, Eftychia Tsironi, Konstantinos Papatheodorou, Dimitrios Nikolakis

**Affiliations:** ^1^Department of Gastrointestinal Endoscopy, Bioclinic of Piraeus, 34 Filellinon Street, 18536 Piraeus, Greece; ^2^Department of Gastroenterology, “Metaxa” Memorial Cancer Hospital of Piraeus, 51 Mpotasi Street, 18537 Piraeus, Greece

## Abstract

Gastrointestinal cytomegalovirus (CMV) infection is a common opportunistic infection in immunocompromised patients, especially patients with acquired immunodeficiency syndrome and transplant recipients. In contrast, CMV infection of the gastrointestinal tract is rare in immunocompetent individuals. We report a case of severe, protracted, and debilitating diarrhea caused by generalized CMV infection of the gastrointestinal tract in an elderly woman with no apparent immunosuppression. An extensive diagnostic investigation demonstrated CMV-associated disease affecting both the upper and lower gastrointestinal tracts (esophagus, small intestine, and colon). Such extensive simultaneous involvement of the alimentary tract in an immunocompetent patient is rare and presents a diagnostic and therapeutic challenge. The diagnosis was based on a combination of endoscopic, histopathological, serological, and polymerase chain reaction analysis findings and our patient was successfully treated with intravenous ganciclovir. Our case demonstrates that gastrointestinal CMV infection should be considered in the differential diagnosis of severe chronic diarrhea in immunocompetent patients and that antiviral treatment may be justified in this setting.

## 1. Introduction

Cytomegalovirus (CMV) is a very prevalent human pathogen with 40–100% of the adult population showing serological evidence of past infection [1]. CMV is excreted in body fluids and mainly transmitted via close personal contact, with most infections being acquired in the perinatal period, infancy, or early adulthood. In immunocompetent hosts primary infection is usually subclinical, although a mononucleosis-like syndrome may occur [1]. After the primary infection, CMV remains latent in the host and can be reactivated later in life. Clinically significant disease in adults, either primary or reactivation, usually occurs in immunodeficient patients, namely, acquired immune deficiency syndrome (AIDS) patients and those receiving chemotherapy, steroids, or immunosuppressive therapy [2]. In contrast, severe CMV disease is rare in immunocompetent hosts.

## 2. Case Presentation

A 71-year-old woman presented with a four-month history of worsening watery diarrhea (up to 10 stools per day) accompanied with progressive weakness, anorexia, and weight loss of approximately 10 kg. Her past medical history included hypertension, depression, and type II diabetes. She had no history of malignancy and no known immunodeficiency disorder, nor was she on any immunosuppressive medication. She did not have any complications related to her diabetes which was well controlled with diet and oral medication (metformin).

On examination, she appeared ill and was lethargic and disoriented. She was severely dehydrated and had marked pitting edema on her legs, significant muscle wasting, small superficial mouth ulcers, and small perineal ulcerations. Diminished breath sounds were noted bilaterally. Her abdomen was distended with mild diffuse tenderness but without any peritoneal signs; increased bowel sounds and shifting dullness were also noted. She was oliguric and hypotensive (systolic blood pressure <100 mm Hg). Initial laboratory investigations revealed significant electrolytic abnormalities (K^+^, 2.5 mmol/L; Na^+^, 129 mmol/L; Ca^2+^, 7.0 mg/dL; P^3+^, 1.6 mg/dL), hypoalbuminemia (2.0 g/dL), mild renal insufficiency (creatinine, 2 mg/dL), and a mild anemia (Hb, 10 g/dL). Chest X-ray revealed bilateral effusions and ultrasound confirmed the presence of ascites.

After aggressive treatment with intravenous fluids combined with albumin infusions, her renal function, diuresis, serum albumin, electrolytes, and blood pressure gradually normalized. On the first day of her hospitalization, a fever of 38.5°C was noted and she was started on empirical antibiotic treatment with metronidazole and ciprofloxacin. During the following week, she continued to have severe diarrhea (>10 bowel movements per day) and a fluctuating pyrexia of up to 38.5°C. She continued to require aggressive supportive care, was unable to tolerate a normal diet, and was started on parenteral alimentation. An extensive diagnostic workup was undertaken during this period to determine the cause of her condition.

**Figure 1:**
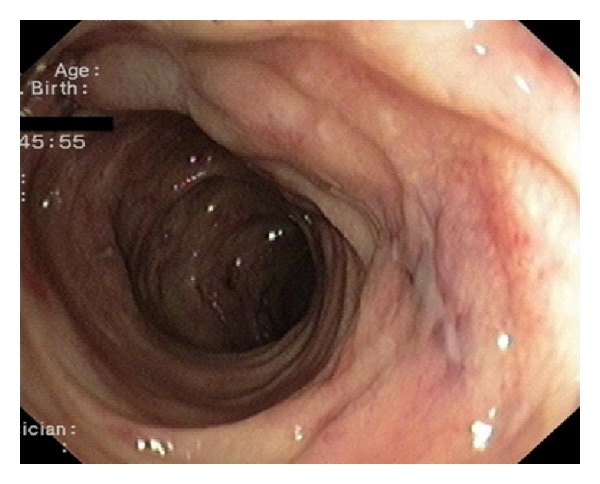
Edema and shallow ulcers in the sigmoid colon. Histology revealed atypical cells with CMV inclusion bodies.

**Figure 2:**
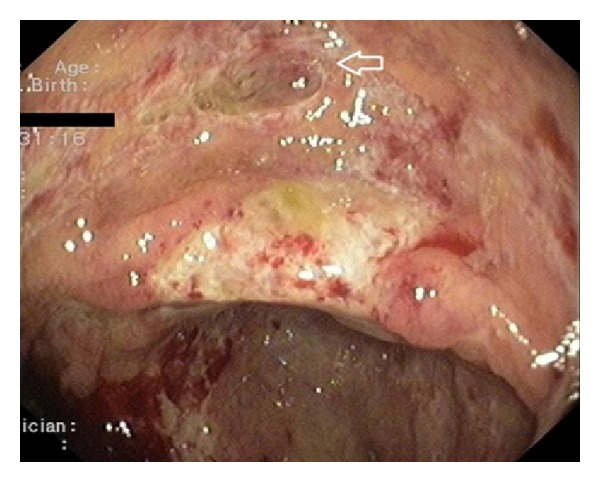
Confluent ulcerations and a “punched-out” ulcer (arrow) in the cecum. Histology revealed atypical cells with possible CMV inclusion bodies.

**Figure 3:**
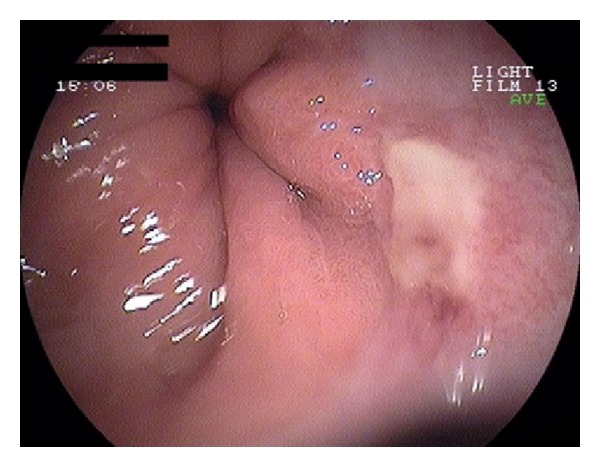
A small superficial ulcer at the gastroesophageal junction. PCR analysis in biopsies was positive for CMV DNA.

**Figure 4:**
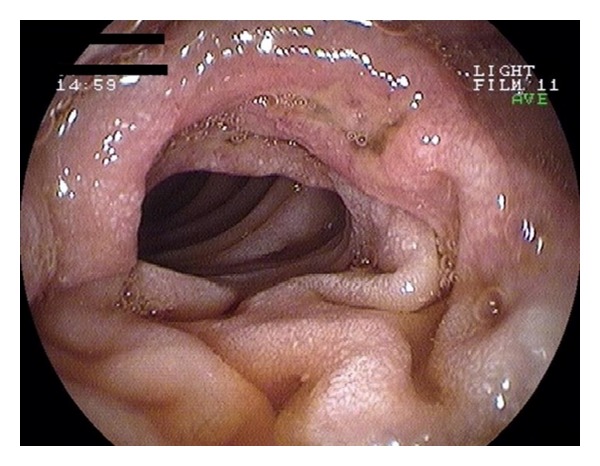
An ulcerated lesion with surrounding edema in the 2nd part of the duodenum. Immunohistochemistry revealed CMV positive cells.

Blood cultures and stool studies (including C. Difficile toxin) were negative. HIV testing was also negative. CT scans showed large bilateral pleural effusions, a medium volume ascites, and thickening of the entire colon wall. Cytology and culture of the ascitic and pleural fluids were negative. Colonoscopy demonstrated edema and multiple small ulcers throughout the left colon (Figure 1). In the right colon, large confluent ulcerations and a few “punched-out” ulcers were noted (Figure 2). Only one small superficial ulcer was found in the terminal ileum. Biopsies from the terminal ileum revealed mild, nonspecific inflammation whereas those from the colon showed granulation tissue, a dense inflammatory infiltrate, and atypical cells with possible CMV inclusion bodies; findings were suggestive of a local vasculitis caused by CMV. Esophagogastroduodenoscopy revealed a small superficial ulcer at the gastroesophageal junction (Figure 3) and a large ulcerated lesion in the descending duodenum (Figure 4). Histology demonstrated mainly granulation tissue but immunohistochemistry revealed CMV positive cells in the duodenal lesion. Capsule endoscopy detected a few small “punched-out” ulcers throughout the small intestine. Polymerase chain reaction (PCR) analysis was positive for CMV DNA in biopsies from the esophageal ulcer and revealed high levels of CMV viremia (9943 copies/mL). Both IgM and IgG anti-CMV antibodies were positive in high titers.

Based on these findings, a diagnosis of CMV infection affecting the esophagus, small intestine, and colon was reached. Antibiotics were stopped and intravenous ganciclovir (5 mg/kg/12 hours) was started. The patient became afebrile within a few days and her condition improved significantly over the next three weeks. Her bowels gradually returned to normal and she was able to tolerate a normal diet allowing discontinuation of the parenteral alimentation. The ganciclovir dose was halved (5 mg/kg/day) after three weeks of treatment. A colonoscopy five weeks after starting antiviral therapy showed significant improvement and ganciclovir was stopped. She was discharged after a total of eight weeks of hospitalization. Her bowel movements were completely normal and she was ambulatory and on a normal diet. Repeating colonoscopy one month after her discharge showed complete healing of all ulcerations. Biopsies from this colonoscopy showed only mild nonspecific inflammation with no histopathological signs of CMV infection. She remains asymptomatic during 4 years of follow-up.

## 3. Discussion

CMV can affect any part of the alimentary tract from the mouth to the anus [2]. Gastrointestinal CMV disease is particularly common in AIDS patients, usually presenting as esophagitis or colitis [3]. In contrast, CMV disease is considered rare in normal hosts. A systematic review identified 290 cases of severe CMV disease in immunocompetent patients. In this review, gastrointestinal tract disease was the most prevalent and it was found in 91 cases (31%) [4]. The colon is the most common site affected in gastrointestinal CMV disease in immunocompetent patients whereas small bowel involvement is less frequent [4].

The symptoms of gastrointestinal CMV disease depend mainly on the site of primary involvement. CMV esophagitis usually causes odynophagia and CMV gastritis commonly presents with epigastric pain [5]. Enterocolitis may manifest with abdominal pain, anorexia, nausea, vomiting, weight loss, watery or bloody diarrhea, hematochezia, and melena [4, 5]. Complications of CMV enterocolitis include massive hemorrhage, megacolon, and perforation and may necessitate surgical intervention [6, 7].

Multiple modalities have been used to diagnose CMV infection. Common endoscopic findings include isolated or multiple ulcers, erosions, and mucosal hemorrhage, although mucosal sloughing, pseudopolyps, pseudomembranes, and even mass lesions in the colon have been observed [8, 9]. Histology is often considered the “gold standard” for diagnosing end-organ disease. Routine stains demonstrate enlarged (cytomegalic) cells with intranuclear inclusions, sometimes surrounded by a clear halo (“owl's eye” effect) [2]. Immunohistochemistry increases the diagnostic yield compared with routine stains [10]. Serology has a limited role as the presence of IgM antibodies suggests active infection but does not establish tissue-invasive disease [2]. Culture has low sensitivity and requires long time to yield results. PCR can detect the CMV DNA in body fluids and tissue specimens with good sensitivity and specificity. Antigen tests that detect the pp65 viral protein can only be used in blood samples [5, 10]. In our patient, end-organ disease was confirmed with positive PCR analysis and/or histopathological evidence of CMV in affected tissues. As mentioned, CMV (like other members of the herpesviridae family) remains latent in the host after the primary infection and can be reactivated later. Our patient's prior CMV serological status was unknown. The concomitant presence of IgG and IgM antibodies probably suggests a reactivation of latent CMV given that past CMV infection is very common in the adult population. However, a de novo infection cannot be excluded as the production of IgG antibodies could have occurred during the many weeks (4 months) of her ongoing illness.

Gastrointestinal CMV can mimic a variety of conditions including ischemic colitis, pseudomembranous colitis, inflammatory bowel disease, and even carcinoma [11]. Crohn's disease and Behcet's disease were considered in our patient due to the clinical presentation, endoscopic findings, and the involvement of multiple gastrointestinal sites. However, the presence of viraemia, the demonstration of CMV in the affected sites, and the excellent response to antiviral treatment effectively excluded these diagnoses. The extended follow-up with no signs of these diseases reaffirmed the diagnosis of CMV.

Our patient did not have any evidence of immunodeficiency. However, aging has been associated with a decline in cell-mediated and humoral immunity possibly making the elderly more susceptible to opportunistic infections like CMV [12].

Although symptomatic CMV infection in immunocompetent patients has been considered to have a benign and self-limited course, an increasing number of severe or even life-threatening cases have been reported, raising questions about the need of specific antiviral treatment in these patients. In a meta-analysis which included 44 cases of CMV colitis, spontaneous remission with supportive treatment occurred in only one-third (31.8%) of all the patients and in 50% of those less than 55 years of age. An overall mortality rate of 31.8% was reported, with a trend towards decreased survival for patients with advanced age (>55), male gender, preexisting immune-modulating comorbidities, and need for colectomy [13]. A much lower mortality rate (6.2%) was noted in 32 new cases of severe gastrointestinal CMV disease identified in a recent review [4]. Two of the 32 patients died, one of which was on antiviral therapy.

Data on the role and effectiveness of antiviral treatment in immunocompetent patients with severe CMV infection are mainly anecdotal as no randomized, controlled trials exist. Physicians generally tend to initiate antiviral therapy in these patients on a case-to-case basis as no specific guidelines have been established. Ganciclovir has been extensively used to treat CMV infection in immunocompromised patients. Side effects include myelosuppression, rash, hypotension, vomiting, diarrhea, and headache. The benefits and risks of antiviral therapy in nonimmunodeficient patients must be weighed carefully before any therapeutic decision is reached [4]. Biopsies from our patient's 1-month follow-up colonoscopy did not reveal any signs of ongoing tissue-invasive CMV infection. There are no data examining the role of oral maintenance antiviral therapy in preventing recurrence of CMV disease in immunocompetent patients and the risk of drug-induced complications probably outweighs the potential benefits of such an approach. In our case we decided to closely follow up the patient and not to initiate maintenance therapy.

Our case demonstrates that although clinically significant gastrointestinal CMV infection is rare in immunocompetent adults, it should be considered in the differential diagnosis of severe chronic diarrhea when more common etiologies have been excluded. A high index of suspicion and the use of various diagnostic modalities may be necessary for making an accurate and prompt diagnosis. Given the fact that high mortality rates have been reported in immunocompetent patients with CMV colitis, especially elderly patients with comorbidities, specific antiviral treatment may be justified in these patients despite the lack of established guidelines.
